# Voltage-Dependent Dopamine Potency at D_1_-Like Dopamine Receptors

**DOI:** 10.3389/fphar.2020.581151

**Published:** 2020-10-07

**Authors:** Richard Ågren, Kristoffer Sahlholm

**Affiliations:** ^1^ Department of Neuroscience, Karolinska Institutet, Stockholm, Sweden; ^2^ Department of Integrative Medical Biology, Umeå University, Umeå, Sweden; ^3^ Wallenberg Centre for Molecular Medicine, Umeå University, Umeå, Sweden

**Keywords:** dopamine D1 receptor (D1R), voltage sensitivity, G protein-coupled receptor, GIRK channels, binding kinetics, G protein selectivity, dopamine D5 receptor (D5R)

## Abstract

In recent years, transmembrane voltage has been found to modify agonist potencies at several G protein-coupled receptors (GPCRs). Whereas the voltage sensitivities of the Gα_i/o_-coupled dopamine D_2_-like receptors (D_2_R, D_3_R, D_4_R) have previously been investigated, the putative impact of transmembrane voltage on agonist potency at the mainly Gα_s/olf_-coupled dopamine D_1_-like receptors (D_1_R, D_5_R) has hitherto not been reported. Here, we assayed the potency of dopamine in activating G protein-coupled inward rectifier potassium (GIRK) channels co-expressed with D_1_R and D_5_R in *Xenopus* oocytes, at -80 mV and at 0 mV. Furthermore, GIRK response deactivation rates upon dopamine washout were measured to estimate dopamine dissociation rate (k_off_) constants. Depolarization from -80 to 0 mV was found to reduce dopamine potency by about 7-fold at both D_1_R and D_5_R. This potency reduction was accompanied by an increase in estimated dopamine k_off_s at both receptors. While the GIRK response elicited via D_1_R was insensitive to pertussis toxin (PTX), the response evoked via D_5_R was reduced by 64% (-80 mV) and 71% (0 mV) in the presence of PTX. Injection of oocytes with Gα_s_ antisense oligonucleotide inhibited the D_1_R-mediated response by 62% (-80 mV) and 76% (0 mV) and abolished the D_5_R response when combined with PTX. Our results suggest that depolarization decreases dopamine affinity at D_1_R and D_5_R. The voltage-dependent affinities of dopamine at D_1_R and D_5_R may be relevant to the functions of these receptors in learning and memory.

## Introduction

Dopamine (DA) receptors are G protein-coupled receptors (GPCRs) subgrouped into the D_1_-like (D_1_R and D_5_R) and the D_2_-like (D_2_R, D_3_R, and D_4_R) receptor families. The D_1_-like receptors are mainly coupled to stimulatory Gα_s/olf_ proteins and expressed in striatum, cortex, amygdala, and hippocampus (Huang et al., 2001; Sarinana et al., 2014) where they are involved in cognitive functions such as memory and attention (Sawaguchi and Goldman-Rakic, 1994; Carr et al., 2017).

D_1_R activation has been closely related to prefrontal cortex functioning by a U-shaped relationship; moderate D_1_R activation is required for optimal performance in learning and memory tasks (Goldman-Rakic et al., 2004; Arnsten et al., 2010). Recent data also implicate D_5_R in working memory and prefrontal cortex function (Carr et al., 2017). Accordingly, D_1_R agonists (many of which are also D_5_R agonists) and positive allosteric modulators are being investigated as putative therapeutics for the treatment of cognitive deficits, e.g., in Alzheimer’s disease, schizophrenia, and Parkinson’s disease (Lewis et al., 2015; Bruns et al., 2018; Luderman et al., 2018; Yang et al., 2018).

Agonist affinities and functional potencies at a number of GPCRs, including D_2_R, D_4_R, and muscarinic M_2_ receptors (M_2_R; (Ben-Chaim et al., 2003; Sahlholm et al., 2008a), have been shown to be regulated by the membrane potential. Interestingly, DA potency in D_3_R-mediated GIRK activation was not significantly different between -80 and 0 mV, suggesting that this receptor might be insensitive to voltage (Sahlholm et al., 2008a). However, the influence of the membrane potential on agonist potency at the D_1_-like family of receptors remains unexplored. Several previous experimental investigations of GPCR voltage-dependence have made use of two-electrode voltage-clamp in *Xenopus* oocytes heterologously expressing GPCRs and G protein-coupled inward-rectifying potassium (GIRK) channels. This has allowed for investigation of GPCR activity by measuring GIRK activation evoked by Gα_i/o_-coupled GPCRs (Ben-Chaim et al., 2003; Sahlholm et al., 2008a; Sahlholm et al., 2011; Sahlholm et al., 2012) and by Gα_q_-coupled GPCRs in the presence of a chimeric Gα_q-i_ protein (Ohana et al., 2006).

Although GIRK channel opening is elicited by Gβγ, which can be associated with a range of Gα subunits, activation of native GIRK currents is mediated almost exclusively via Gα_i/o_- and not Gα_s/olf_-proteins. This specificity may be achieved through the higher rate of turnover of the G protein cycle of Gα_i/o_-proteins, liberating higher local concentrations of Gβγ upon receptor activation (Touhara and MacKinnon, 2018). However, based on previously published work with similarly G_s_-coupled β-adrenergic receptors (Lim et al., 1995; Wellner-Kienitz et al., 2001; Hatcher-Solis et al., 2014), we speculated that high expression levels of D_1_R and D_5_R in *Xenopus* oocytes might allow their downstream Gα_s/olf_ proteins to release sufficient amounts of Gβγ to activate GIRK channels, thus allowing us to investigate the putative voltage sensitivities of these receptors. Importantly, previous work has shown that neither G protein dissociation into Gα and Gβγ, nor GIRK activation by Gβγ, are intrinsically voltage dependent processes, thus making GIRK currents a suitable readout for studies of GPCR voltage sensitivity (Ben-Chaim et al., 2003). Here, we report the differential potencies of DA in D_1_R- and D_5_R-mediated GIRK activation at -80 and 0 mV.

## Methods

### Molecular Biology

Wildtype human D_1_R (from Dr. Marc Caron, Duke University, NC), D_5_R (from the cDNA Resource Center, Bloomsberg, PA; www.cdna.org) were in pcDNA3.1 while PTX-S1 cDNA (from Dr. Eitan Reuveny, Weizmann Institute of Science, Israel) was in pGEM-HE. GIRK1 (Kir3.1) and GIRK4 (Kir3.4) (GenScript, Piscataway, NJ) cDNA were in pXOOM (provided by Dr. Søren-Peter Olesen, University of Copenhagen, Denmark). Plasmids were linearized using the appropriate restriction enzymes (D_1_R, D_5_R; XhoI, PTX-S1; Nhe1 and GIRK1/GIRK4; NotI), followed by in vitro transcription using the T7 mMessage mMachine kit (Ambion, Austin, TX). cRNA concentration and purity were determined by spectrophotometry.

### Oocyte Preparation

Oocytes from the African clawed toad, *Xenopus laevis*, were isolated surgically as described previously (Sahlholm et al., 2011). The surgical procedures have been approved by the Swedish National Board for Laboratory Animals and the Stockholm Ethical Committee. Following 24 h of incubation at 12°C, oocytes were injected with 4.5 ng D_1_R receptor cRNA or 25.5 ng D_5_R receptor cRNA and 50 pg of each GIRK1 and GIRK4 cRNA, using the Nanoject II (Drummond Scientific, Broomall, PA) and a volume of 50 nl per oocyte. When used, 3 ng PTX-S1 cRNA was injected, based on previous observations of complete inhibition of D_2_R-induced GIRK activation with this amount of cRNA (Agren et al., 2018a). In a subset of experiments, 10 pmol/oocyte (in a volume of 50 nl) of an antisense DNA oligonucleotide (sequence: GCTCATATTGGCGCAGGTGCAT) directed against *X. laevis* Gα_s_ mRNA was injected 48 h before electrophysiology recordings. This treatment has previously been described to abolish G_s_-dependent signaling in oocytes (de la Pena et al., 1995).

### Electrophysiology Methods 

Following cRNA injection into oocytes and 7 days of incubation at 12°C, electrophysiological experiments were conducted using the parallel eight-channel, two-electrode voltage-clamp, OpusXpress 6000A (Molecular Devices, San Jose, CA). Continuous perfusion was maintained at 1.5 ml/min. Data were acquired at membrane potentials of -80 mV or 0 mV and sampled at 134 Hz using the OpusXpress 1.10.42 (Molecular Devices) software. To increase the inward rectifier potassium channel current at negative potentials, a high potassium extracellular buffer was used (in mM: 64 NaCl, 25 KCl, 0.8 MgCl_2_, 0.4 CaCl_2_, 15 HEPES, 1 ascorbic acid, adjusted to pH 7.4), yielding a K^+^ reversal potential of about -40 mV. In experiments with 1 mM KCl, the NaCl concentration was 88 mM. DA was purchased from Sigma-Aldrich (St. Louis, MO). Recordings were performed at room temperature (22 °C).

### Data Analysis 

Electrophysiological data were analyzed in Clampfit 10.6 (Molecular Devices). Concentration-response curves were fitted using least squares nonlinear regression in GraphPad Prism 8 (GraphPad Software, San Diego, CA). For each oocyte and each holding voltage, the current response to each concentration of DA tested was normalized to the response to the maximally effective concentration of DA at the same voltage and in the same oocyte. The following equation was fitted to the normalized agonist data:

$$Y=Bottom+(1-Bottom)/(1+10^{(\log EC_{50}-X)\times n})$$

where *Y* is the normalized response, *X* the logarithm of DA concentration, and *n* the Hill slope.

The washout decay time constant, τ_off_, was obtained from single exponential fits (using Levenberg-Marquardt least-squares fitting in Clampfit 10.6) to the washout-induced current deactivation time course. The responses to 1 µM (D_1_R) and 100 nM (D_5_R) DA were used for analysis of response deactivation kinetics. The first 10 s following agonist washout were discarded, and the exponential function was fit to the data over ~70% of the response amplitude. k_off_ was calculated from τ_off_ using the following relation:

$$k_{off}=\frac{1}{\tau_{off}}$$

Data are represented as mean ± SEM. Concentration-response data were analyzed by comparing the fractional responses to DA at individual concentrations at -80 mV and at 0 mV using Student’s paired t-test or, if data were not normally distributed, Wilcoxon signed rank test. Normality was assessed using the Shapiro-Wilk test. Current amplitudes were compared using one-way ANOVA with Tukey’s multiple comparisons test or, if data were not normally distributed, Kruskal-Wallis test with Dunn’s multiple comparisons test. k_off_ rates were compared using the Wilcoxon signed rank test. The significance threshold was p<0.05. Statistical analyses were performed in GraphPad Prism 8.

## Results

The effects of DA application on membrane currents were investigated in oocytes co-expressing D_1_R or D_5_R with GIRK1/4 channels. DA was found to elicit inward currents at -80 mV, whereas at 0 mV, outward currents were observed. No appreciable current response to DA could be recorded in oocytes expressing D_1_R or D_5_R in the absence of GIRK channels at either voltage (**Supplementary Figure 1**), nor did DA elicit any response in oocytes expressing GIRK channels without D_1_R or D_5_R (not shown).

**Figure 1:**
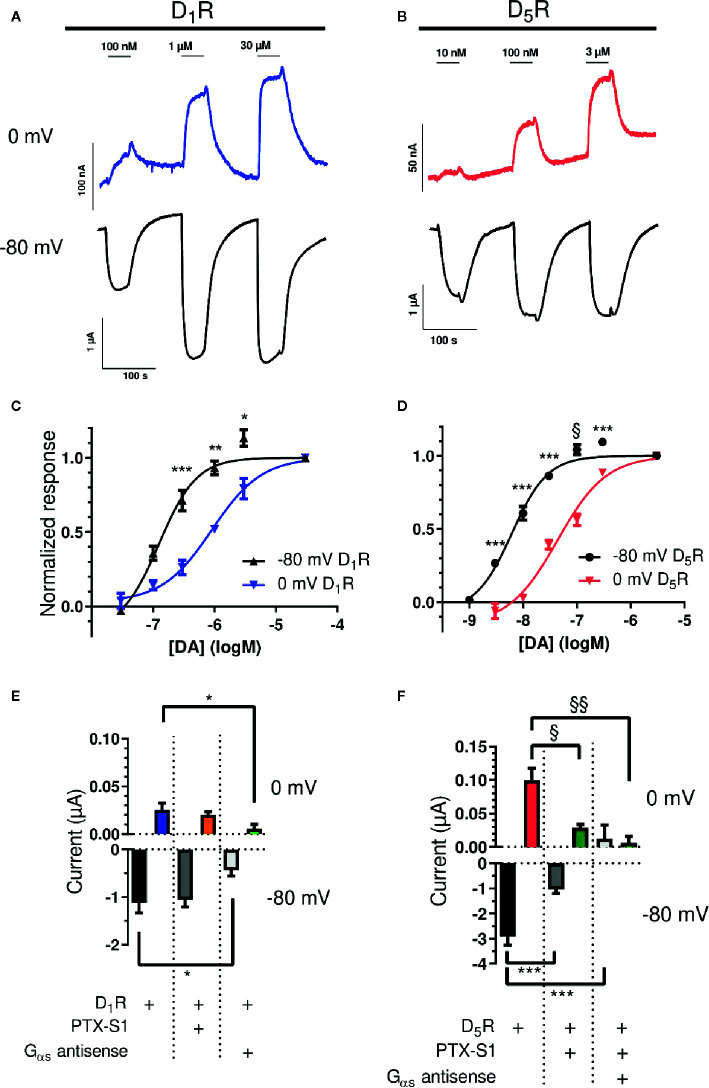
Voltage-sensitive potency and G protein-dependence of GIRK activation by D_1_-like receptors. **(A)** Representative traces of D_1_R-evoked GIRK currents upon application and washout of 100 nM, 1, and 30 µM DA at 0 (blue) and -80 mV (black). **(B)** Representative traces of D_5_R-evoked GIRK currents upon application and washout of 10 nM, 100 nM, and 3 µM DA at 0 (red) and -80 mV (black). **(C)** Concentration-response curves for D_1_R-evoked GIRK current at -80 mV and 0 mV. The pEC_50_ for DA was 6.902 ± 0.120 (125 nM; Hill slope 1.419 ± 0.348) and 6.043 ± 0.078 (906 nM; Hill slope 1.074 ± 0.165) at -80 mV and 0 mV, respectively (n = 3 - 5). *p < 0.05, **p < 0.01, ***p < 0.001, Student’s paired t-test. **(D)** Concentration-response curves for D_5_R-evoked GIRK current at -80 mV and 0 mV. The pEC_50_ for DA was 8.211 ± 0.065 (6 nM; Hill slope 1.301 ± 0.166) and 7.361 ± 0.078 (44 nM; Hill slope 0.959 ± 0.116) at -80 mV and 0 mV, respectively (n = 6). ***p < 0.001, Student’s paired t-test, ^§^p < 0.05, Wilcoxon signed rank test. Curves were obtained by fitting a variable-slope sigmoidal function to the data using least squares nonlinear regression (see Methods). **(E)** Mean currents (± SEM) evoked by 10 µM DA in oocytes expressing D_1_R and GIRK1/4 with or without PTX-S1 or G_αs_ antisense oligonucleotide at 0 mV (top) and -80 mV (bottom). At -80 mV, mean currents were -1.12 ± 0.21 µA (D_1_R; n = 7), -1.06 ± 0.15 µA (+PTX-S1; n = 7), and -0.43 ± 0.13 µA (+G_αs_ antisense oligonucleotide; n = 6). At 0 mV, mean currents were 0.025 ± 0.007 µA (D_1_R; n = 7), 0.020 ± 0.003 µA (+PTX-S1; n = 7), and 0.006 ± 0.004 µA (+G_αs_ antisense oligonucleotide; n = 7). *; p<0.05, Tukey’s multiple comparisons test. **(F)** Mean currents (± SEM) evoked by 10 µM DA in oocytes expressing D_5_R and GIRK1/4 with or without PTX-S1, and G_αs_ antisense oligonucleotide at 0 mV (top) and -80 mV (bottom). At -80 mV, mean currents were -2.92 ± 0.35 µA (D_5_R; n = 7), -1.05 ± 0.16 µA (+PTX-S1; n = 6), and 0.012 ± 0.021 µA (+PTX-S1+G_αs_ antisense oligonucleotide; n = 3). Note that in the presence of antisense oligonucleotide, there was a small deflection in positive direction during DA application. At 0 mV, mean currents were 0.100 ± 0.018 µA (D_5_R; n = 7), 0.029 ± 0.005 µA (+PTX-S1; n = 6) and 0.006 ± 0.010 µA (+PTX-S1+G_αs_ antisense oligonucleotide; n = 4). ***p < 0.001, Tukey’s multiple comparisons test. ^§^p < 0.05, ^§§^p < 0.01, Kruskal-Wallis test with Dunn’s multiple comparisons test.

DA potency at D_1_-like receptors was investigated by DA applications (40-s applications, each followed by 100-s washout) of increasing concentration to oocytes injected with D_1_R or D_5_R and GIRK1/4 cRNA. Submaximally effective concentrations were applied, followed by a maximally effective concentration (30 µM for D_1_R and 3 µM for D_5_R), at both -80 and 0 mV (**Figures 1A, B**). At D_1_R, the DA EC_50_ was 125 nM at -80 mV, increasing significantly to 906 nM at 0 mV (**Figure 1C**), a potency shift of about 7-fold. The DA EC_50_ at D_5_R was 6.1 nM at -80 mV, again increasing significantly to 44 nM at 0 mV (**Figure 1D**), resulting in a ~ 7-fold decrease in DA potency. The current–voltage relationships of the membrane currents, as assessed by 4-s ramps and normalized to the amplitude at -80 mV, were virtually superimposable in the absence and in the presence of DA (**Supplementary Figure 2**).

To assess whether GIRK-activation was mediated via Gα_i/o_ proteins, the catalytic subunit of pertussis toxin (PTX-S1), which inactivates Gα_i/o_ proteins by ADP-ribosylation, was expressed in the oocytes (Vivaudou et al., 1997). In addition, oocytes were injected with an antisense oligonucleotide designed to knock down Gα_s_ expression (de la Pena et al., 1995). Co-expression of PTX-S1 with D_1_R and GIRK1/4 did not significantly affect GIRK response amplitudes to 10 µM DA, neither at -80 mV nor at 0 mV. However, injection of the Gα_s_ antisense oligonucleotide strongly and significantly suppressed the DA-evoked current response by 62% (-80 mV) and 76% (0 mV; **Figure 1E**). In contrast, in oocytes expressing D_5_R and GIRK1/4, the amplitudes of GIRK responses to 10 µM DA were significantly reduced by 64% and 71% compared to control at -80 and 0 mV, respectively, when PTX-S1 was introduced. With the further addition of the Gα_s_ antisense oligonucleotide, the DA response was virtually abolished (**Figure 1F**).

**Figure 2:**
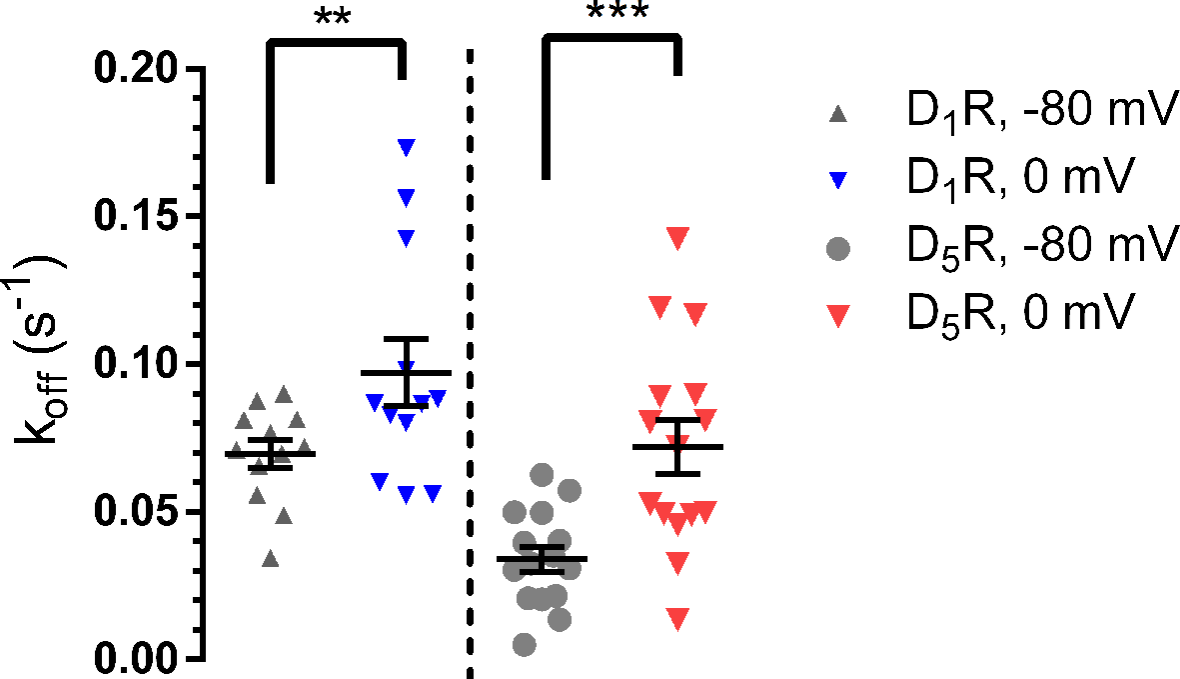
Current deactivation rate upon DA washout. Following depolarization, the deactivation rate increased at the D_1_R (-80 mV; 0.070 ± 0.005 s^-1^, 0 mV; 0.097 ± 0.011 s^-1^; n = 12 for both potentials; p = 0.0049, Wilcoxon signed rank test) and D_5_R (-80 mV; 0.034 ± 0.004 s^-1^, 0 mV; 0.072 ± 0.009 s^-1^; n = 15 for both potentials; p < 0.001, Wilcoxon signed rank test) following depolarization. Horizontal bars indicate means ± SEM. **, p = 0.0049; ***, p < 0.001.

The rate of GIRK response deactivation upon removal of agonist has been used to approximate the time course of agonist dissociation from its receptor (Bunemann et al., 2001; Benians et al., 2003) and changes in the rates of GIRK deactivation between hyperpolarized and depolarized potentials have previously been shown to reflect reciprocal increases or decreases in agonist dissociation rates and consequently, affinities (Ben-Chaim et al., 2003; Ohana et al., 2006; Ben Chaim et al., 2013). Exponential functions were fitted to the time courses of GIRK current deactivation upon DA washout (see *Methods*). The rate of response decay was observed to increase, both at D_1_R and at D_5_R, when the membrane was depolarized from -80 mV to 0 mV (**Figure 2**). As judged by the rate of current increase (at -80 mV) upon wash-in of buffer containing 25 mM KCl from a baseline reading in buffer containing 1 mM KCl, the rate of solution exchange was faster than the fastest rate of decrease of the GIRK response upon DA washout (**Supplementary Figure 3**).

Finally, to visualize the change in DA potency when stepping from one voltage to another, we performed experiments in oocytes co-expressing D_1_R with GIRK1/4 channels where a 40-s prepulse to between -80 to 0 mV was followed by a -80 mV post-pulse, in the absence or presence of an intermediately- (300 nM) or a maximally (30 µM) effective concentration of DA. Subtracting the basal, agonist-independent current from the current evoked in the presence of agonist, a slow current increase at -80 mV was evident following depolarized prepulses in the presence of 300 nM, but not 30 µM DA (**Supplementary Figure 4**). This behavior of GIRK currents, which has been referred to as “relaxation”, has earlier been demonstrated to be a consequence of receptor voltage sensitivity and to reflect the increase in agonist binding to the receptor, at submaximally effective concentrations, upon hyperpolarization of the membrane (Moreno-Galindo et al., 2011; Sahlholm, 2011).

## Discussion

Previous investigations have found evidence for reduced agonist potencies and affinities at depolarized potentials in several Gα_i/o_-coupled GPCRs, including M_2_R, D_2_R, histamine H3 and H4, and the metabotropic glutamate 3 receptor (mGluR3) (Ben-Chaim et al., 2003; Ben-Chaim et al., 2006; Sahlholm et al., 2008b; Sahlholm et al., 2012), as well as the Gα_s_-coupled β-adrenergic receptors (Birk et al., 2015). Conversely, a number of Gα_q_-coupled muscarinic, metabotropic glutamate, and prostanoid receptors demonstrated increased agonist potencies at depolarized potentials (Ben-Chaim et al., 2003; Ohana et al., 2006; Kurz et al., 2020). The potency shifts of 7-fold between -80 and 0 mV observed here for D_1_R and D_5_R are among the more pronounced shifts reported so far.

Compared to the D_2_-like DA receptors previously investigated (Sahlholm et al., 2008a), D_5_R and D_1_R present the strongest reductions in DA potency (~7-fold) between -80 and 0 mV, whereas D_2_R showed a somewhat smaller potency reduction of about 4-fold. D_4_R and D_3_R were the least sensitive, with ~2-fold and 1.1-fold (non-significant) lower DA potency at 0 compared to -80 mV (Sahlholm et al., 2008a). DA concentrations in the striatum vary between the low nanomolar range at extrasynaptic sites (corresponding to tonic DA signaling), to high micromolar concentrations in the immediate vicinity of active DA terminals (phasic DA signaling) (Marcott et al., 2014; Bamford et al., 2018). Assuming the in vivo EC_50_s of D_1_R and D_5_R to be similar to those observed here, one would expect the voltage sensitivity of D_1_R to be more relevant during phasic DA signaling, when DA concentrations transiently reach sufficiently high concentrations to activate this receptor. Conversely, the influence of voltage over D_5_R signaling might be more pronounced under tonic DA signaling conditions, which would be expected to produce a sub-saturating D_5_R response. Given the involvement of both D_1_R and D_5_R in learning and memory, it is tempting to speculate that their sensitivity to voltage may enable these receptors to function as a form of coincidence detectors, reporting on both the membrane potential and the presence of DA. For example, strong depolarization of the postsynaptic neuron may spatially restrict the signaling of D_1_-like receptors by allowing for efficient activation only of those receptors which are located sufficiently close to active DA terminals.

Overexpression of G_s_-coupled receptors has been reported to support activation of GIRK channels, although much less efficiently than G_i/o_-coupled receptors, in several expression systems including *Xenopus* oocytes (Lim et al., 1995; Wellner-Kienitz et al., 2001; Hatcher-Solis et al., 2014). Thus, we believe that the PTX-insensitive D_1_R-mediated GIRK activation observed here is likely to be elicited via G_s_ signaling, although this phenomenon may be a consequence of receptor overexpression and unlikely to take place in native tissue. Indeed, this conclusion is strengthened by the observation that injection of an antisense oligonucleotide directed towards *X. laevis* mRNA encoding Gα_s_ strongly reduced the current response to DA in oocytes co-expressing D_1_R and GIRK channels.

Interestingly, in contrast to the D_1_R-evoked responses, a major component of the GIRK currents elicited upon D_5_R stimulation was PTX-sensitive, suggesting that D_5_R is able to activate G_i/o_ proteins in addition to G_s/olf_. While D_5_R-mediated G_q_ signaling has been observed in some heterologous systems (So et al., 2009), G_i/o_-coupling of D_5_R has, to the best of our knowledge, not been reported previously. In *Xenopus* oocytes, G_q_-mediated calcium signaling would typically elicit a characteristic, rapidly desensitizing response mediated through endogenous calcium-activated chloride channels (Hansen and Bräuner-Osborne, 2009); however, we did not observe any such responses upon D_5_R activation.

Contrary to the present observations, D_1_R has previously been reported to couple to PTX-sensitive G_o_ proteins in addition to G_s_, while D_5_R was found to couple to the PTX-insensitive inhibitory G protein, G_z_ (Sidhu et al., 1998), which efficiently activates GIRK (Vorobiov et al., 2000). However, available evidence suggests that there is no detectable endogenous G_z_ expression in *Xenopus* oocytes (Vorobiov et al., 2000; Kalinowski et al., 2003), making it unlikely that G_z_ activation underlies the PTX-insensitive component of D_5_R-mediated GIRK activation observed here. Instead, it appears more likely that this component is G_s_-mediated. Again, this assumption is strengthened by the abolition of the DA-induced current response by injection of Gα_s_ antisense oligonucleotide into oocytes co-expressing D_5_R and GIRK with PTX-S1.

The depolarization-induced decrease in acetylcholine potency at the M_2_R has been related to a corresponding increase in acetylcholine k_off_ at the M_2_R, and a consequent increase in GIRK response deactivation rate at depolarized potentials (Ben-Chaim et al., 2003; Ben Chaim et al., 2013; Agren et al., 2018b; López-Serrano et al., 2020). Similar findings have been reported for the mGluR3 and histamine H_3_ receptors (Ohana et al., 2006; Sahlholm et al., 2012). Likewise, in the present study, we found the rates of GIRK current deactivation upon DA washout to be increased at 0 mV compared to -80 mV. Presumably, these changes in deactivation rates reflect faster DA k_off_s at D_1_R and D_5_R at 0 mV.

Finally, it should be emphasized that the oocyte expression system does not represent a native environment for D_1_R and D_5_R, which interact with a host of other proteins in neurons and other tissues. However, this heterologous system provides a well-defined background with no detectable expression of endogenous DA receptors which lends itself well to stable voltage-clamp recordings and detection of even relatively small agonist-induced responses with a high signal-to-noise ratio. The DA EC_50_s for D_1_R- and D_5_R-induced GIRK activation at 0 mV in the present study agree fairly well with the reported values for high-affinity DA binding in isolated membranes in the literature, recapitulating the higher affinity of D_5_R (24 nM; Weinshank et al., 1991) compared to D_1_R (324 nM; Marcellino et al., 2012), suggesting that the DA binding characteristics of these receptors in oocytes are similar to those of D_1_R and D_5_R expressed in mammalian cells.

## Conclusion

The present results reveal that DA potency at D_1_R and D_5_R is decreased upon depolarization from -80 to 0 mV. Our interpretation is that DA k_off_ rate constants are affected by membrane voltage and contribute to a decrease in DA affinity upon depolarization. This dependence of D_1_R and D_5_R-mediated responses upon transmembrane voltage could allow these receptors, which have been implicated in learning and memory, to function as a sort of “coincidence detectors”, responding robustly to low concentrations of DA only at hyperpolarized potentials.

## Data Availability Statement

The raw data supporting the conclusions of this article will be made available by the authors, without undue reservation.

## Ethics Statement

The animal study was reviewed and approved by Swedish National Board for Laboratory Animals, Stockholm Ethical Committee (Stockholms djurförsöksetiska nämnd).

## Author Contributions

RÅ and KS designed the experiments, RÅ conducted the experiments, RÅ and KS analyzed data, RÅ drafted the first version of the manuscript. KS supervised the project. All authors contributed to the article and approved the submitted version.

## Funding

This study was supported by grants from Stiftelsen Lars Hiertas Minne, Åhlénstiftelsen, and Magnus Bergvalls stiftelse (to KS). KS is currently a fellow at the Wallenberg Center for Molecular Medicine at Umeå University.

## Conflict of Interest

The authors declare that the research was conducted in the absence of any commercial or financial relationships that could be construed as a potential conflict of interest.
