# Supplementary material for: Voltage-Dependent Dopamine Potency at D1-Like Dopamine Receptors
Source: Front Pharmacol. 2020 Oct 7;11:581151. doi: 10.3389/fphar.2020.581151 (PMC7577048; doi:10.3389/fphar.2020.581151)
Supplement: Supplementary file 1 [file DataSheet_1.docx]

**Supplementary Figure 1.** Absence of DA-evoked current response at -80 and 0 mV, as indicated, in oocytes expressing (**A**) D_1_R and (**B**) D_5_R in the absence of GIRK channels. Horizontal bars indicate period of DA application.





**Supplementary Figure 2.** Normalized current-voltage relationships for basal and DA-evoked GIRK currents in oocytes co-expressing D_1_R or D_5_R with GIRK1/4 channels. (**A**) Oocytes expressing D_1_R and GIRK1/4 channels were recorded during continuous ramps between +20 and -80 mV in high-potassium buffer (25 mM KCl) in the presence or absence of 30 µM DA. n = 5 oocytes. (**B**) Oocytes expressing D_5_R and GIRK1/4 channels were recorded during continuous ramps between +20 and -80 mV in high-potassium buffer (25 mM KCl) in the presence or absence of 3 µM DA. n = 6 oocytes. Currents were normalized to the maximal amplitude evoked at -80 mV. Data is presented as means ±SEM, but the error bars are in most cases smaller than the symbols.





**Supplementary Figure 3.** Comparison of solution exchange-mediated and agonist washout-induced GIRK current rates of change. Green bar: Exchange of extracellular buffer from low-K^+^ (1 mM KCl) to high-K^+^ (25 mM KCl) induced a rapid current increase in cells expressing GIRK1/4 (rate of increase; 0.251 ± 0.125 s^-1^, n = 8). Dark grey, blue, light grey, and red bars: Deactivation rates of the DA-evoked GIRK response upon DA washout at -80 and at 0 mV in oocytes co-expressing D_1_R or D_5_R with GIRK1/4 channels, as indicated, are shown for comparison (same data as shown in Figure 2). Data are shown as mean ± SEM.


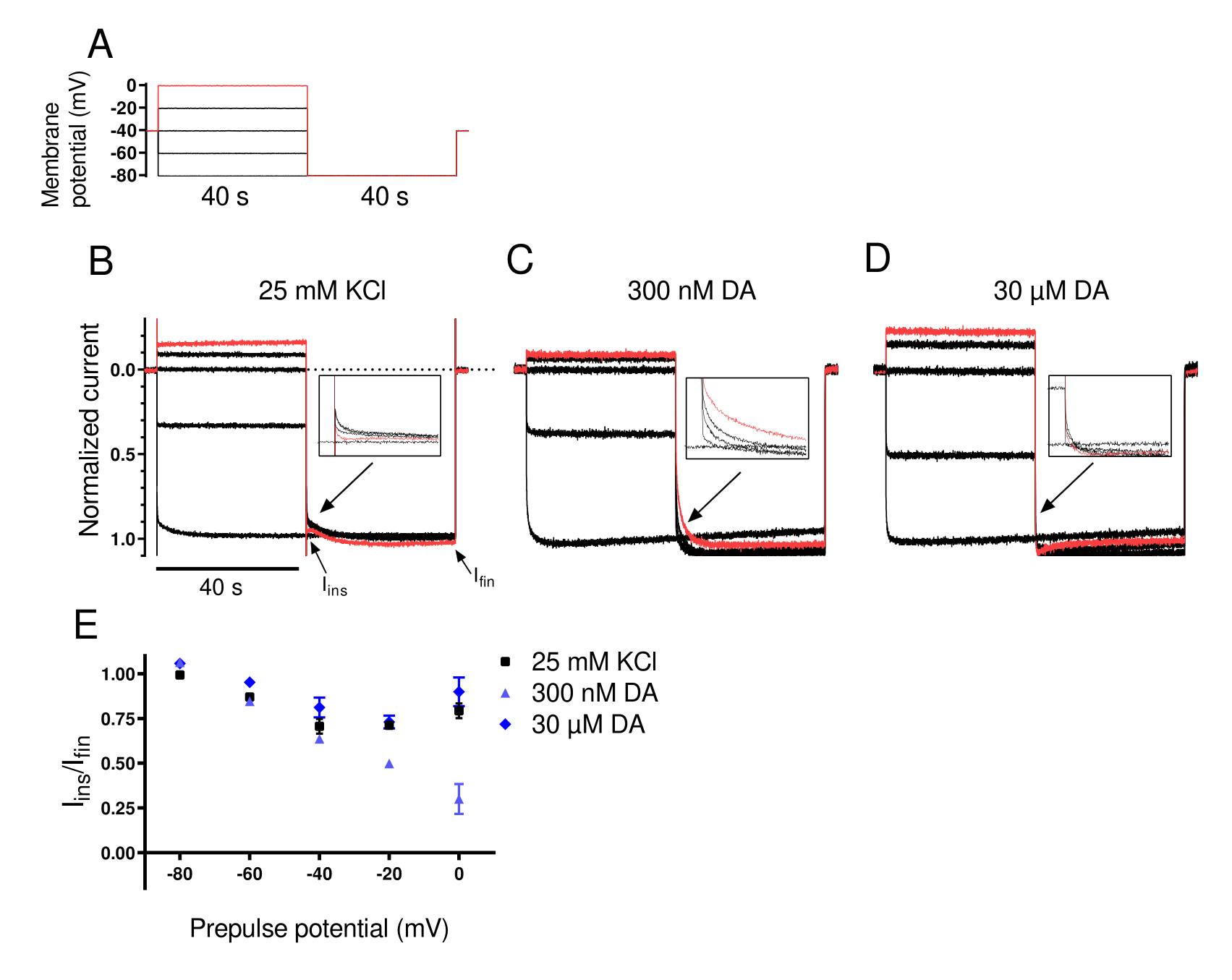


**Supplementary Figure 4.** DA-dependent GIRK current relaxation. (**A**) Voltage protocol consisting of a variable-voltage prepulse and a -80 mV post-pulse. (**B**) Currents evoked by the protocol in (**A**) in an oocyte co-expressing D_1_R and GIRK1/4 channels in high-K^+^ (25 mM KCl) recording buffer in the absence of agonist. (**C**) The same cell recorded 60 s after application of an intermediately effective (300 nM) concentration of DA. (**D**) The same oocyte 60 s following application of a saturating concentration (30 µM) of DA. In both (**C**) and (**D**), the current traces shown in (**B**) have been subtracted to reveal the agonist-induced currents. Insets cover the last 0.5 s of the prepulse and first 2.5 s of the post-pulse. The y axis projection of the insets corresponds to 0.4 to 1.1. The first trace in each recording, with a 0 mV prepulse, is highlighted in red. In each recording (**B**, **C**, and **D**), the currents have been normalized to the final current amplitude (I_fin_) of the first trace. (**E**) Ratio of instantaneous (I_ins_) to final GIRK current amplitudes during the -80 mV post-pulse, as indicated in (**B**), following prepulses to the indicated potentials. 25 KCl; n = 7, 300 nM DA; n = 3, 30 µM DA; n = 3.
